# Supplementary material for: High sedation needs of critically ill COVID-19 ARDS patients—A monocentric observational study
Source: PLoS One. 2021 Jul 27;16(7):e0253778. doi: 10.1371/journal.pone.0253778 (PMC8315516; doi:10.1371/journal.pone.0253778)
Supplement: S1 Table — (DOCX) [file pone.0253778.s001.docx]

**S1 Table:** Applied sedatives and analgetic dosages. Data presented as mean (SD). Administered continuous intravenous drug dose as a single or multiple application. Sedatives were always combined with the administration of an opioid.

| **substance** | **dosing unit** | **overall average** | **single use sedative** | **combination of two sedatives** | **combination of three sedatives** |
| --- | --- | --- | --- | --- | --- |
|  |  | **dosage** | **dosage** | **dosage** | **dosage** |
| sedatives: |  |  |  |  |  |
| clonidine | µg kg^-1^ h^-1^ | 1.54 (0.79) | 1.49 (0.71) | 1.59(0.71) | 1.81 (0.75) |
| midazolam | mg kg^-1^ h^-1^ | 0.14 (0.11) | 0.12 (0.07) | 0.13 (0.06) | 0.22 (0.20) |
| esketamine | mg kg^-1^ h^-1^ | 0.86 (0.76) | - | 0.57 (0.61) | 1.25 (0.65) |
| propofol | mg kg^-1^ h^-1^ | 1.66 (1.40) | 2.73 (0.93) | 2.29 (0.98) | 2.38 (0.93) |
| dexmedetomidine | µg kg^-1^ h^-1^ | 0.54 (0.58) | 1.02 (0.20) | 1.06 (0.45) | 0.66 (0.52) |
| lormetazepam | mg kg^-1^ min^-1^ | 0.013 (0.023) | 0.021 (0.011) | 0.019 (0.030) | 0.017 (0.010) |
| analgetics: |  |  |  |  |  |
| sufentanile | µg kg^-1^ h^-1^ | 0.13(0.09) | 0.11(0.06) | 0.13(0.09) | 0.23(0.12) |
| remifentanile | µg kg^-1^ min^-1^ | 0.15(0.05) | - | 0.15(0.00) | 0.16(0.07) |

Abbreviations: µg= micrograms, mg= milligrams, kg= kilograms bodyweight, h= hour, min= minute.
